# A Comprehensive Comparison of PICSI and ICSI Techniques Through a Triple-Blinded Trial: Effects on Embryo Quality, Cumulative Pregnancy Rate, and Live Birth Rate

**DOI:** 10.3390/biomedicines13051104

**Published:** 2025-05-01

**Authors:** Lucia Alegre, Laura Carrión-Sisternas, Lorena Bori, Irene Hervás, Jose Remohí, Nicolás Garrido, Marcos Meseguer

**Affiliations:** 1 IVIRMA Global Research Alliance, IVIRMA Valencia, Plaza de la Policía Local 3, 46015 Valencia, Spain; 2 IVIRMA Global Research Alliance, IVI Foundation, Instituto de Investigación Sanitaria La Fe (IIS La Fe), 46026 Valencia, Spain; laura.carrion@ivirma.com (L.C.-S.); lorena.bori@ivirma.com (L.B.); irene.hervas@ivirma.com (I.H.); nicolas.garrido@ivirma.com (N.G.)

**Keywords:** PICSI, sperm selection, hyaluronic acid binding, oocyte donation, embryo quality, cumulative pregnancy rate, live birth rate

## Abstract

**Background**: Sperm selection is critical in assisted reproduction, typically relying on swim-up and centrifugation density gradients. New methods, such as PICSI (physiological intracytoplasmic sperm selection), aim to enhance outcomes by selecting mature sperm based on hyaluronic acid (HA) binding and have generated interest due to their potential impact on the clinical outcomes of patients who undergo assisted reproductive treatments. **Methods**: A single-center, prospective, and triple-blinded study was conducted with 277 couples in the egg donation program. The oocytes of each recipient patient were randomly microinjected using the ICSI or PICSI technique and maintained in culture in time-lapse incubators until blastocyst formation. Biological and clinical outcomes were analyzed, including fertilization and blastocyst formation rates, embryo morphokinetics, pregnancy, miscarriage, and live birth rates, and artificial intelligence-assigned embryo quality scores. **Results**: Clinical outcomes were comparable between the two groups, but a higher pregnancy rate was observed in the PICSI group than in the ICSI group (74.04% vs. 70.87%). Although blastocyst formation rates were similar on both day 5 (D5) and day 6 of development, the proportion of good-quality embryos on D5 was higher in the PICSI group (68.27%) than in the ICSI group (63.47%) (*p* > 0.05). Finally, the cumulative pregnancy rate was significantly higher in the experimental group than in the control group (88% vs. 72%) after four embryo transfers (*p* < 0.05). **Conclusions**: Utilizing HA to perform sperm selection during ICSI procedures does not increase live birth rates. However, it may enhance the quality of the selected sperm. This could be beneficial for patients in egg donation programs, particularly for those who have experienced repeated pregnancy loss.

## 1. Introduction

The use of assisted reproductive technologies (ART) has risen substantially in recent years, with nearly half a million treatments performed annually in a population of 300 million—equivalent to an average of 1581 cycles per million inhabitants [1]. Among these procedures, intracytoplasmic sperm injection (ICSI) stands out as the predominant technique. Originally developed in 1992 to address cases of severe male factor infertility [2], ICSI has since become a routine practice in many fertility clinics, even in the absence of male factor indications [3]. During the ICSI procedure, sperm selection is primarily based on morphology and motility; however, this approach does not reliably exclude immature or chromosomally abnormal spermatozoa [4,5,6], which can negatively impact the outcomes of assisted reproductive treatments [5,7].

A more detailed analysis of the semen sample prior to insemination may facilitate the selection of the most competent spermatozoa, ultimately improving clinical outcomes and enhancing the overall efficiency of each assisted reproduction cycle [8,9]. To this end, several sperm selection techniques have been developed to better assess sperm quality and ensure the selection of the most optimal sperm for fertilization [10,11]. In recent years, the MACS (magnetic-activated cell sorting) technique has become one of the most studied strategies for advanced sperm selection. This method is based on immunomagnetic cell sorting, targeting sperm that externalize phosphatidylserine in their plasma membrane, a characteristic marker of the onset of apoptosis [12,13]. The technique employs microspheres coated with annexin V, a protein with a high affinity for phosphatidylserine, allowing the retention of apoptotic spermatozoa on a magnetic column and resulting in a fraction enriched in viable and functionally competent spermatozoa [13]. Another advanced sperm selection technique developed to select optimal spermatozoa or sperm with higher DNA integrity is the hypo-osmotic swelling test (HOST). The molecular basis of this methodology is that mature, functional spermatozoa with an intact plasma membrane show a characteristic pattern of flagellar movement, especially under conditions of osmotic stress, as occurs in hypo-osmotic solutions [14]. Another method, intended solely for intracytoplasmic microinjections of spermatozoa because of its individual selection, is IMSI—MSOME (motile sperm organellar morphology examination), where spermatozoa are selected based on the morphology of their acrosome, post-acrosomal lamina, midpiece, nucleus, and the presence of vacuoles, using a microscopy system at high magnification (up to 6600×). Together with MSOME, the PICSI or physiological ICSI technique has also been widely investigated for its ability to select mature and functionally competent spermatozoa immediately prior to their microinjection into oocytes [10]. HA is a glycosaminoglycan that is part of the extracellular matrix of the cumulus oophorus and zona pellucida of the female gamete, and the binding of it to specific receptors present in the sperm cell membrane is essential once the acrosomal reaction occurs in vivo [15,16]. Therefore, if we transfer this phenomenon to the in vitro fertilization (IVF) laboratory, spermatozoon–oocyte binding can be imitated. 

The use of sperm selected based on their ability to bind to hyaluronic acid (HA) has attracted considerable interest due to its potential impact on clinical outcomes in patients undergoing assisted reproductive treatments. However, the results reported in the literature remain controversial. While some studies have demonstrated improvements in various laboratory and clinical parameters, such as fertilization rate [17,18,19], embryo quality, live birth rate, or miscarriages [15,20,21,22,23], other investigations have found no significant differences in clinical outcomes when comparing HA-based sperm selection to the conventional ICSI method [9,24,25,26,27].

In all the studies published to date, there is no homogeneity in terms of the clinical results obtained and the study population. However, in our study, PICSI has been evaluated as a method of sperm selection in couples included in the oocyte donation program, eliminating the female factor as a possible bias that may interfere with the conclusions. To our knowledge, no previous studies with the same design as ours have been identified in the most recent literature. The objective is to demonstrate whether the PICSI technique, as a sperm selection method, increases the live birth rate and improves the results of in vitro fertilization techniques in couples in the egg donation program.

## 2. Materials and Methods

### 2.1. Study Design

The trial presented here is a prospective, randomized, triple-blind cohort study conducted at the IVI-RMA Valencia clinic. For the inclusion of the patients in the study, the signing of informed consent was required. This study was approved by the Ethics Committee of the Research Center (1310-VLC-134-LA; September 2015).

**Figure 1:**
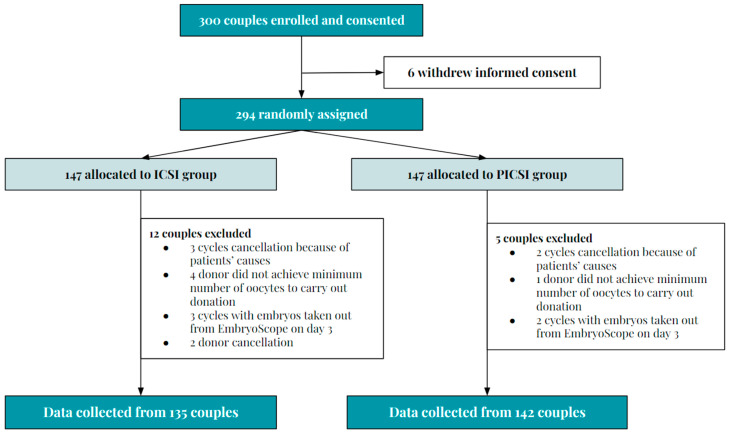
Flow of the patient recruitment process. Initially, 300 patients were offered participation in this study, ensuring that the established sample size was reached. After the refusal of informed consent by 6 couples, 294 were randomly assigned to the ICSI (n = 147) and PICSI (n = 147) groups. After the exclusion of 12 couples in the ICSI group and 5 in the PICSI group (for the reasons stated in the figure), data were collected from 135 couples in the ICSI group and 142 in the PICSI group.

Patients who agreed to participate and who did not withdraw consent were included in a randomized, computer-assigned manner into two study groups: the PICSI group or the ICSI group. In the ICSI group (control group), 12 couples were excluded because of different reasons, such as cycle cancellations due to patient-related causes, insufficient oocytes for ovodonation treatment, or donor cancellation, among others (Figure 1). In the PICSI group, five couples were excluded (Figure 1).

### 2.2. Study Population

Data were collected from a total of 277 infertile couples undergoing their first ICSI cycle within an oocyte donation program, using fresh ejaculated semen and either fresh or frozen-thawed embryo transfer. Eligible female recipients were required to be under 45 years of age, have a body mass index (BMI) below 30 kg/m^2^, and have no major uterine malformations. All oocytes used were from anonymous donors, either fresh or vitrified. The mean age of oocyte donors was approximately 25 years, with a maximum of 35 years. Only fresh semen samples with a sperm concentration greater than 5 million/mL were included. Cases using frozen semen or testicular biopsy-derived sperm were excluded. Additionally, couples with a history of recurrent miscarriage (defined as two or more pregnancy losses) were not included in this study.

### 2.3. Sample Size Determination and Study Blinding

This study was conducted at the Assisted Reproduction Unit of the IVI Valencia clinic, where, as of 2014, 1500 in vitro fertilization cycles with donated oocytes were performed annually. We estimated that 90% of these cycles met the inclusion and exclusion criteria for participation in this study. The proportion of patients achieving clinical pregnancy and subsequent live births in the donor oocyte population was approximately 55%.

Our hypothesis was that the use of PICSI, as demonstrated by Worrilow et al. (2013) [20], increased the likelihood of pregnancies resulting in live births by 15%. Using the macro Macro!N2IP V2006.02.24 (JM. Domenech, R. Granero, and R. Sesma) SAMPLE SIZE and POWER DETERMINATION: two independent proportions, the required sample size per group is 128 patients per study arm, with an alpha risk of 5% and a beta risk of 20%, equivalent to a statistical power of 80%. The calculation method follows a normal asymptotic approximation and a one-sided hypothesis. We estimate a 5% loss of patient inclusion. Therefore, a total of 270 patients (135 in the control group and 135 in the study group) will be required to complete the study under the proposed hypothesis. Recruitment costs are more than sufficient to achieve the necessary sample size.

The triple-blind design ensures that the patients, the embryologists assessing fertilization and embryo development, and the statisticians conducting the analysis were not aware of the study group to which each patient belonged. Only the embryologist who performed the microinjection identified each patient as group A or group B. The equivalence of groups A and B with the control or experimental group was revealed after analysis.

### 2.4. Sperm Sample Collection and Preparation

Seminal samples were collected on the day of microinjection by masturbation into sterile containers after 2–5 days of sexual abstinence. After an incubation period of 10 min at 37 °C and 6% CO_2_, they were analyzed to determine sperm concentration and motility using a Makler chamber (Sefi Laboratories, Tel Aviv, Israel). Morphology analysis was carried out using the Bio-DiffTM stain (Biognost, Zagreb, Croatia) and adhered to the World Health Organization’s (WHO, 2021) current criteria. Subsequently, sperm selection was carried out using swim-up as described by Meseguer et al. (2024) [28].

### 2.5. Controlled Ovarian Stimulation and Transvaginal Oocyte Retrieval

Ovarian stimulation of donors and oocyte retrieval was performed as described by Bori et al. (2022) [29]. In summary, a gonadotropin-realizing hormone agonist was administered to donors until more than eight follicles had reached a mean diameter superior to 18 mm. Oocyte retrieval was performed 36 h later through follicular aspiration. Oocytes were first washed in a gamete medium (Cook Medical, Australia) and then cultured in a fertilization medium (Origio, Cooper Surgical, Denmack) at 5% CO_2_, 5% O_2_, and 37 °C. After 4 h, mechanical and chemical denudation of follicles was carried out just before ICSI.

### 2.6. Oocyte Microinjection: ICSI vs. PICSI

Metaphase II (MII) oocytes were microinjected using either the conventional ICSI or the PICSI technique. For standard ICSI, sperm selection was based on motility and morphology, as assessed by the embryologist under an Olympus IX7 microscope with 400x optical magnification prior to injection. Sperm selection and immobilization were performed on a plastic dish containing drops of culture medium and polyvinylpyrrolidone (PVP; 10% solution in a carbonated medium), overlaid with mineral oil, and maintained at 37 °C. Selected spermatozoa were captured, immobilized, and subsequently microinjected into mature oocytes.

For PICSI, sperm selection was performed using specialized hyaluronic acid (HA)-coated crystal plates (Sperm Selection Device^®^, Origio, CooperSurgical, Denmark). The HA spots were rehydrated with 10 μL of Fert medium (Origio, CooperSurgical, Denmark), followed by the addition of approximately 10 μL of the processed semen sample. After a 5–10 min incubation period, spermatozoa that exhibited head binding to HA and active tail movement were selected. Between 10 and 15 HA-bound sperm were then transferred to PVP drops for morphological assessment. Those that met the morphological criteria were immobilized and microinjected into the oocytes following the same protocol as conventional ICSI.

Following microinjection, oocytes from both the ICSI and PICSI groups were cultured in pre-equilibrated EmbryoSlides (Vitrolife, Göteborg, Sweden), each containing 16 microwells filled with 90 μL of single-step culture medium (Gems; Genea Biomedx, Rowville, Australia) and overlaid with 1.6 mL of mineral oil.

### 2.7. Embryo Incubation, Scoring, and Selection

Injected oocytes were incubated in an Embryoscope TM (ES) time-lapse incubator (Vitrolife, Viby, Denmark) with a gassed culture medium. Conditions remained stable throughout all embryonic development: 37 °C, 6% CO_2_, and 5% O_2_. Fertilization was confirmed 16–18 h after insemination, with the presence of two pronuclei (PN) and two polar corpuscles. Embryos were kept in incubators until day 5 (D5) or day 6 (D6) of development, depending on the most appropriate transfer date. Embryo development was assessed on an external computer with software for the analysis (EmbyoViewer workstation v2.0; Vitrolife). To evaluate and select blastocysts, a hierarchical classification process was used that combined two criteria: the standard Association for the Study of Reproductive Biology (ASEBIR) morphologic grading, which assesses the inner cell mass (ICM) and the trophectoderm cells (TE) [30] (Supplementary Tables S1–S4), and the KIDScore D5 V3.2 algorithm, which studies the kinetic parameters provided by the time-lapse technology (EmbryoViewer software v2.0; Vitrolife). Among good-quality embryos, the best embryo was chosen for transfer (fresh cycle) and the rest were vitrified for a possible later deferred cycle. The first criterion for embryo selection was the ASEBIR category. If two or more embryos were assigned the same category, the one with the highest score was selected.

### 2.8. Pregnancy Determination and Clinical Pregnancy and Live Birth Rates

Biochemical pregnancy was determined with a blood analysis of the receiving woman 11 days post-transfer, and it was confirmed with a result of beta-human chorionic gonadotropin >10 IU/L. Clinical pregnancy was subsequently confirmed with gestational sac visualization and heartbeat detection 21–23 days after transfer. Clinical miscarriage was determined as gestational sac absence, by ultrasound, before 12 weeks. Finally, the live birth rate (LBR) was obtained with the birth of one or several babies at the end of the pregnancy.

### 2.9. Statistical Analysis

Statistical tests were applied to prove significant differences in the values of each variable. All statistical calculations were performed using IBM SPSS 25 (Statistical Package for the Social Sciences; IBM Corp, Armonk, NY, USA). A statistically significant result was considered with a *p*-value < 0.05. Firstly, a descriptive analysis of the patients was performed, including the mean as the main parameter and an ANOVA test to check if there were significant differences between the population of the study group (PICSI) and the control group (ICSI). This was achieved by analyzing and comparing key variables such as patient age, donor age, BMI, the number of oocytes aspirated and microinjected, etc. The ANOVA test was also used in this study to compare the morphokinetic parameters of the embryos in each group. The rest of the parameters were statistically analyzed using contingency tables and Pearson’s chi-square test. This applied to both fresh and delayed cycles (devitrified embryo transfer cycles). In addition, two binary logistic regression analyses were performed, one simple and one multivariate, including several confounding variables.

## 3. Results

### 3.1. Study Population Characteristics

A total of 277 couples were recruited; these were randomly assigned to the PICSI group (n = 142) or ICSI group (n = 135). A total of 3104 microinjected oocytes, 2433 zygotes, 1144 viable embryos (transferred and vitrified), 348 fresh transferred embryos, 140 devitrified transferred embryos, and 203 live newborns were generated. The characteristics of the subjects who participated and the clinical results obtained were similar in both groups (Table 1).

An average of 11 MII oocytes per recipient were obtained for microinjection in both groups. The fertilization rate was 68.62% in the PICSI group versus 77.72% in the ICSI group (*p* < 0.001), although the mean fertilization rate per recipient was not statistically different between the ICSI and PICSI groups (8.84% vs. 7.59%) (Table 1). Furthermore, non-statistically significant differences were found between haploidy (0.90% vs. 0.36%) and triploidy (0.21% vs. 0.24%) fertilization failure, the mean number of useful embryos per patient (four for both groups), or the number of embryos transferred (1.61 vs. 1.63) (*p* > 0.05) (Table 1). In terms of semen sample characteristics, both sperm concentration and sperm motility were similar in both study groups (Table 1).

### 3.2. Embryo Development and Embryo Quality

Embryos were cultured until day 5 or 6 of development, corresponding to the blastocyst stage. On day 5, although no statistically significant differences were observed between the PICSI and ICSI groups, a higher proportion of embryos had reached the blastocyst stage in the control (ICSI) group (63.03% vs. 58.12%, *p* > 0.05). Similarly, no significant differences were detected in the distribution of advanced embryonic stages—cavitated blastocyst (CB), expanded blastocyst (EB), initiating hatching blastocyst (iHB), and hatched blastocyst (HB)—between the two groups on either day 5 or day 6 (see Supplementary Table S5). The cumulative blastocyst formation rate (day 5 + day 6) was 60.64% in the PICSI group and 63.89% in the ICSI group (*p* > 0.05).

Regarding embryo quality, a higher proportion of good-quality embryos (grades A and B) was observed on day 5 in the PICSI group (68.27%) compared to the ICSI group (63.47%), although this difference did not reach statistical significance (*p* > 0.05). Likewise, the proportion of non-viable embryos was slightly lower in the PICSI group (2.84%) compared to the control group (3.79%), but again without statistical significance (*p* > 0.05). A similar pattern was noted on day 6, with the PICSI group presenting a higher percentage of good-quality embryos (35.50%) relative to the ICSI group (30.77%). Additionally, the ICSI group exhibited a greater proportion of non-viable embryos on day 6 (27.94%) than the PICSI group (23.81%), although this difference was also not statistically significant (*p* > 0.05).

In addition to morphological parameters, we evaluated whether differences in morphokinetic variables existed that were associated with key developmental milestones. Statistically significant differences were found between groups. Pronuclear appearance occurred earlier in the PICSI group compared to the ICSI group (8.80 h vs. 9.14 h, *p* < 0.05). Conversely, the time taken to reach the eight-cell stage was significantly shorter in the ICSI group than in the PICSI group (66.61 h vs. 90.94 h, *p* < 0.05). Interestingly, initiation of blastocyst formation was observed earlier in the PICSI group (101.55 h) relative to the control group (102.59 h, *p* < 0.05), whereas the onset of hatching occurred significantly earlier in the ICSI group (110.90 h) compared to the PICSI group (114.77 h, *p* < 0.05).

Finally, embryo quality at the blastocyst stage was also retrospectively evaluated using iDAScore, an AI-based scoring system developed through deep learning, which assigns a numerical value to embryos based on time-lapse videos and images of their morphokinetic development. The mean iDAScore of viable embryos (i.e., those suitable for transfer or vitrification) was identical between groups (ICSI: 5.28 ± 7.44; PICSI: 5.28 ± 7.44; *p* > 0.05). Likewise, the mean iDAScore of embryos selected for fresh transfer did not differ significantly between the ICSI (6.21 ± 2.62) and PICSI (6.20 ± 2.67) groups (*p* > 0.05), suggesting comparable predicted implantation potentials across conditions.

### 3.3. Clinical Results: Pregnancy Rate; Miscarriage Rates, and Live Birth Rate

With regard to clinical results, the LBR was similar between both groups, with 52.88% of newborns in the PICSI group and 57.28% in the control group (*p* > 0.05) for fresh cycles. The same occurred in cycles with devitrified embryo transfer (Table 2). We found the same trend for the pregnancy rate; however, the rate of pregnancy loss (biochemical and clinical miscarriage) was higher in the PICSI group (21.15%) than in the ICSI group (13.59%) (*p* > 0.05) for fresh cycles, although it was similar for deferred cycles (19.40% vs. 21.00%; *p* > 0.05). Nevertheless, the clinical miscarriage rate was very similar in both fresh groups (Table 2) (Supplementary Table S6).

To further explore the potential influence of the microinjection technique on clinical outcomes, a binary logistic regression analysis was conducted, incorporating the type of insemination procedure (PICSI vs. ICSI) as the primary independent variable. The model aimed to assess its association with key reproductive endpoints, namely clinical pregnancy, miscarriage, and live birth rates, but no statistically significant associations were identified. These findings suggest that the likelihood of achieving pregnancy, experiencing pregnancy loss, or achieving a live birth was not significantly influenced by the type of microinjection technique employed (Table 3).

Next, despite being a prospective study controlled for certain confounding variables, we perform a multivariate logistic regression in order to observe whether any of these confounding variables, related to oocyte quality, seminal sample quality, and the intrinsic characteristics of ICSI cycles, had any effect on the results of the previous binary logistic regression. However, the results obtained from the multivariate logistic regression did not indicate a correlation between any of the variables and clinical outcomes, with the exception of the age of the recipient patient, which correlated with an increased probability of miscarriage in deferred cycles (Table 4).

**Figure 2:**
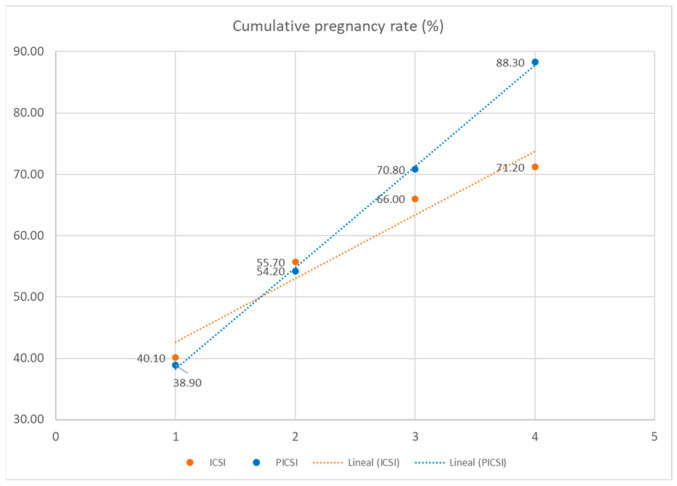
Cumulative pregnancy rate (%).

Finally, we studied the cumulative pregnancy rate according to the number of embryos transferred. We found that the pregnancy rate increases with a higher number of embryos transferred, being similar when the number is lower than three. However, after four embryo transfers, the cumulative pregnancy rate was significantly higher in the PICSI group than in the ICSI group, at 88.30% vs. 71.20%, respectively (Figure 2). Therefore, global cycle efficiency was improved with the PICSI technique.

## 4. Discussion

PICSI is a sperm selection technique designed to facilitate a more targeted evaluation of the semen sample, thereby improving the quality of sperm selected for microinjection into the oocyte [31].

Numerous studies have examined the potential benefits of PICSI as a sperm selection method, yet findings remain diverse and, at times, contradictory. Four major reviews have addressed this controversy. A 2014 Cochrane review, which included only one randomized controlled trial (RCT), concluded that the evidence was insufficient to determine the effect of PICSI on IVF outcomes [9]. In 2016, Beck-Fruchter et al. conducted a meta-analysis, including two RCTs and three sibling oocyte studies, reporting no significant differences in clinical outcomes, although an improvement in embryo quality was observed with PICSI [32]. Similarly, a 2018 review by Avalos-Durán et al. found no significant differences in clinical outcomes, emphasizing the heterogeneity across studies. This variability is largely attributed to differences in the clinical parameters assessed and the characteristics of the studied populations, which hinder the ability to draw unbiased conclusions [25,32]. The most recent Cochrane review, published in 2019 by Lepine et al., included eight RCTs and concluded that the only clinical parameter with a consistent benefit from PICSI was a reduction in miscarriage rate when compared to conventional ICSI [33].

Among all outcome measures in assisted reproductive cycles, the live birth rate (LBR) is considered the most clinically relevant. According to the present biometric analysis, PICSI does not confer a statistically significant advantage regarding the LBR compared to standard ICSI, a finding consistent with previously published data [21,26,34]. For example, a multicenter RCT conducted by Miller et al. (2019) included a large number of couples and concluded that PICSI does not lead to an increase in the LBR [26]. Conversely, some studies have reported a significant improvement in the LBR when fertilization was performed using HA-selected spermatozoa [21,22,23]. These findings were primarily observed in subgroups of older women (>35 years), who typically present with reduced oocyte quality and were identified as the population most likely to benefit from PICSI [23]. In contrast, the present study includes patients from an egg donation program, where the use of high-quality donor oocytes may mitigate the contribution of sperm-related factors to clinical outcomes. This is attributed to the oocyte’s intrinsic ability to repair sperm DNA fragmentation [35,36], which may obscure the potential benefit of PICSI. Thus, the initial hypothesis—using donor oocytes to eliminate the influence of the female factor and isolate the effect of sperm quality—may need to be reconsidered.

In our study, we have not found significant differences in the rest of the clinical results assessed. The pregnancy rate was very similar in both groups, both in fresh embryo transfer cycles and in deferred transfer cycles. These results are in keeping with those previously published, although many of them also obtained significant results [27,37,38,39]. Similarly, Emirdar et al. (2023) conducted a retrospective analysis of 2415 ICSI and 400 PICSI cycles, involving a total of 1630 patients, and found comparable results [27]. However, a study by Scaruffi et al. (2022) reported that PICSI led to an increase in pregnancy and implantation rates in patients with previous failed ICSI cycles [40].

Despite the pregnancy loss rate (biochemical and clinical miscarriage) being higher in the PICSI group than in the ICSI group, the clinical miscarriage rate was similar between both groups. Additionally, the binary and multivariate logistic regression did not find any correlation between PICSI and miscarriage outcomes. Previously published research reported a decrease in miscarriage rates in the PICSI group [20,22,26,34], as did the latest Cochrane review [33] and ESHRE’s report [31]. The potential protective effect of PICSI against spontaneous miscarriage remains controversial. However, it is possible that this technique’s ability to select mature sperm—those with lower DNA damage and fewer aneuploidies—plays a key role in this observed effect [31,41]. This hypothesis would coincide with what was previously discussed for the LBR, so that by using high-quality oocytes, microinjection of sperm with different levels of DNA fragmentation may have no effect thanks to the oocytes’ repair mechanisms.

Regarding laboratory indicators such as the fertilization rate, recent studies, such as those by Kim et al. (2020), Liu et al. (2019), and Novoselsky et al. (2021) [17,18,19], have suggested that PICSI improves fertilization rates compared to ICSI. In contrast, our findings showed a lower fertilization rate in the PICSI group [17,18,19]. This discrepancy highlights the considerable heterogeneity in the results of studies evaluating the PICSI technique. Additionally, we found a non-significant increase in the proportion of good-quality embryos on D5 and D6 when using the PICSI technique compared to ICSI, as previously described in the literature [15,18,19]. Similar results were reported by Scaruffi et al. (2022), who observed improvements in clinical parameters, such as cleavage rate and embryo quality, in patients with prior ICSI failures [40], as did Kim et al. (2020), who studied patients with severe teratozoospermia (<1% normal sperm) [18]. However, when embryo quality was assessed using iDAScore V2.0—a deep learning algorithm trained with time-lapse videos of embryos with known implantation outcomes—identical results were found between embryos formed using PICSI and ICSI. This algorithm assigns a score to each embryo, which correlates with its likelihood of implantation and pregnancy [29]. As such, the probability of successful implantation appears to be very similar for embryos created by PICSI and those created by ICSI.

Finally, we observed an improvement in the efficiency of IVF cycles in patients where sperm selection was carried out using HA. Thus, while West et al. (2022) found that the cumulative pregnancy rate does not improve in patients in the PICSI group, we have indeed found an increase compared to ICSI patients [23]. This finding suggests that PICSI enhances the long-term success of assisted reproduction treatments compared to conventional methods. However, this conclusion should not be interpreted rigidly for several reasons. Notably, despite the higher cumulative pregnancy rate observed in the PICSI group, we did not detect an increase in blastocyst formation rates or a higher proportion of good-quality embryos on day 5, raising questions about the direct causality of this improvement. Furthermore, pregnancy is a multifactorial process influenced not only by sperm quality but also by the intrinsic reproductive characteristics of each woman, such as BMI, smoking or stress [42,43], endometrial receptivity [44], and maternal age [45]. Nevertheless, our study has some strengths as it applies rigorous inclusion criteria, selecting patients under 45 years old with a BMI below 30 kg/m^2^ and no severe uterine malformations. We found no significant differences between the patients in both groups. Furthermore, it is important to recognize that minor changes in reproductive success, which may not be evident in a single cycle, can lead to significant outcomes over multiple cycles or embryo transfers, as could be the case in this study.

A recent ESHRE report discusses indications and suitable populations of patients who could benefit from the PICSI technique [31]. These include couples of advanced age [23] and individuals with a medical history of recurrent miscarriages and/or a lack of live births in previous cycles [19,40], as well as those with abnormal seminal parameters and high sperm DNA fragmentation [31,46]. To better understand the relationship between sperm’s ability to bind to hyaluronic acid, sperm DNA quality, and clinical outcomes, large-scale studies targeting these patient groups are needed [31]. Such studies would help identify the most suitable populations for the PICSI technique.

The limitations of our study include its small sample size (277 couples) and its single-center design, which may limit statistical significance and broader applicability. Additionally, a sibling oocyte cohort study could have provided a better design, where half of the oocytes would be microinjected using the conventional ICSI technique and the other half with PICSI. While our initial hypothesis used donated oocytes to eliminate female factors, we now also consider that the oocyte’s ability to repair sperm DNA defects might mask the possible effects of the PICSI technique in terms of the main laboratory indicators, such as fertilization or blastocyst formation rates, as well as embryo quality. However, our study also has some strengths because we minimized potential confounding factors by applying strict inclusion and exclusion criteria. Moreover, the randomized nature of our study ensured that potential confounding variables were equally distributed between the two groups, reducing bias and enhancing the reliability of our findings. Finally, to eliminate inter-embryologist variability, a single embryologist with three years of experience in ART (prior to the start of the study) performed all PICSI and ICSI procedures, ensuring consistency throughout this study.

## 5. Conclusions

Our study leads us to conclude that sperm selection with hyaluronic acid does not improve live birth rates compared to the conventional method. However, the overall efficiency in cycles where sperm selection and microinjection were performed using the PICSI technique was higher (cumulative rates), at least with the sperm selection device used in this study.

Given these findings, while hyaluronic acid-based sperm selection may not directly enhance live birth rates, its potential benefits should not be overlooked. The observed increase in cumulative pregnancy rates suggests that this method could contribute to improved reproductive outcomes over multiple cycles. Therefore, its use may still be advantageous in specific contexts, such as egg donation programs, particularly in cases of recurrent pregnancy failure. Nonetheless, it is crucial to consider the broader spectrum of factors that ultimately determine reproductive success.

## Figures and Tables

**Table 1:** Study population characteristics. There were non-significant differences between the PICSI and ICSI groups (ANOVA). *p* = *p*-value; *NS* = non-significant differences.

	ICSI (n = 135)		PICSI (n = 142)		
		95% CI		95% CI	*p*
Female age (years) (mean)	40	39.38–40.62	39.76	39.23–40.30	*NS*
Male age (years) (mean)	41.64	40.54–42.73	42.12	41.17–43.06	*NS*
Donor age (years) (mean)	25.68	24.93–26.42	25.54	24.81–26.27	*NS*
BMI (kg/m^2^) (mean)	22.84	22.16–23.51	22.24	21.78–22.70	*NS*
Oocytes aspirated (mean)	13.66	13.26–14.06	13.1	12.73–13.48	*NS*
Oocytes MII inseminated by ICSI (mean)	11.31	11.12–11.50	11.03	10.83–11.23	*NS*
Fertilization Rate (m/rw) ^1^	77.72	75.43–80.01	68.62	65.70–71.09	*NS*
Fertilization Failure (1 PN) (m/rw) ^1^	0.29	0.19–0.39	0.36	0.26–0.45	*NS*
Fertilization Failure (3 PN) (m/rw) ^1^	0.21	0.13–0.29	0.24	0.15–0.34	*NS*
Total fresh sperm (millions) (m/rw) ^1^	86.68	73.16–100.2	78.91	66.58–91.25	*NS*
Fresh sperm concentration (millions/mL) (m/rw) ^1^	46.51	42.06–50.97	44.45	39.26–49.65	*NS*
Fresh sperm motility (%) (m/rw) ^1^	31.22	29.57–32.87	35.11	33.30–36.92	*NS*
Total capacitated sperm (millions) (m/rw) ^1^	1.53	1.18–1.88	2.16	1.4–2.91	*NS*
Embryo rate (vitrificated/transferred) (m/rw) ^1^	4	3.9–4.1	4	3.91–4.09	*NS*
Transferred embryos (m/rw) ^1^	1.61	1.57–1.65	1.63	1.59–1.67	*NS*

^1^ (m/rw = mean/receiving woman).

**Table 2:** Clinical results in cycles with fresh embryo transfer and cycles with devitrified embryo transfer. *F* = fresh embryo transfer cycles; *DF* = deferred embryo transfer cycles. There were non-significant differences between PICSI and ICSI groups. *NS* = non-significant differences.

		PICSI	ICSI	*p*
Pregnancy rate (%)	*F*	74.04	70.87	*NS*
	*DF*	51.50	50.00	*NS*
Pregnancy loss rate (miscarriage rate) (%)	*F*	21.15	13.59	*NS*
	*DF*	19.40	21.00	*NS*
Biochemical miscarriage rate (%)	*F*	8.65	1.94	*NS*
	*DF*	5.10	6.09	*NS*
Clinical miscarriage rate (%)	*F*	12.50	11.65	*NS*
	*DF*	14.29	12.17	*NS*
Live birth rate (%)	*F*	52.88	57.28	*NS*
	*DF*	30.40	26.00	*NS*

**Table 3:** Binary logistic regression results for the probability of gestation, miscarriage, and RNV, according to microinjection technique (PICSI vs. ICSI). *F* = fresh cycles; *DF* = delayed cycles. OR = odds ratio; CI = confidence interval.

	*PREGNANCY*		*MISCARRIAGE*		*LIVE BIRTH*	
	OR	95%CI	OR	95%CI	OR	95%CI
*F*	1.099	0.603–2.002	1.527	0.763–3.058	0.833	0.486–1.425
*DF*	1.529	0.769–3.037	1.039	0.451–2.394	1.564	0.706–3.464

**Table 4:** Multivariate logistic regression analysis. The analysis included the technique used for the oocyte microinjection and some confounder variables. (*): *p* < 0.05.

*PREGNANCY*				
	OR	IC95%	OR	IC95%
PICSI/ICSI	1.620	0.769–3.413	1.576	0.733–3.389
Patient age	1.070	0.938–1.221	1.071	0.945–1.213
Donor age	0.989	0.920–1.063	0.998	0.916–1.087
BMI	1.014	0.932–1.104	1.103	0.998–1.220
Day transfer	1.043	0.944–1.151	1.031	0.946–1.125
Total embryos obtained	0.951	0.251–3.608	1.982	0.917–4.283
Total fresh sperm	0.997	0.991–1.003	1.003	0.995–1.011
Total selected sperm	0.983	0.862–1.121	0.932	0.754–1.152
Number of transferred embryos	1.384	0.673–2.848	0.611	0.263–1.418
*MISCARRIAGE*				
	OR	IC95%	OR	IC95%
PICSI/ICSI	1.109	0.465–2.645	1.089	0.434–2.730
Patient age	1.098	0.933–1.292	1.179 (*)	1.009–1.378
Donor age	0.997	0.917–1.084	0.934	0.840–1.039
BMI	1.033	0.934–1.142	1.045	0.936–1.166
Day transfer	1.039	0.929–1.162	0.975	0.886–1.073
Total embryos obtained	2.476	0.305–20.076	0.907	0.366–2.248
Total fresh sperm	0.996	0.989–1.004	1.001	0.991–1.010
Total selected sperm	1.004	0.880–1.146	0.977	0.762–1.253
Number of transferred embryos	0.757	0.311–1.840	0.633	0.242–1.655
*LIVE BIRTH*				
	OR	IC95%	OR	IC95%
PICSI/ICSI	0.755	0.425–1.340	1.587	0.658–3.828
Patient age	0.959	0.868–1.060	0.930	0.803–1.078
Donor age	0.997	0.941–1.056	1.070	0.96–1.1180
BMI	0.981	0.916–1.050	1.032	0.923–1.154
Day transfer	0.929	0.851–1.016	1.062	0.969–1.164
Total embryos obtained	1.065	0.383–2.963	2.363	0.908–6.153
Total fresh sperm	1.000	0.996–1.004	1.003	0.994–1.012
Total selected sperm	1.066	0.945–1.204	0.956	0.765–1.195
Number of transferred embryos	0.579	0.324–1.034	0.780	0.303–2.004

## Data Availability

All scientists wishing to access the raw data will be granted full and immediate access.

## Supplementary Materials

The following supporting information can be downloaded at: https://www.mdpi.com/article/10.3390/biomedicines13051104/s1, Supplementary Table S1. Categories and characteristics of embryonic inner cell mass according to ASEBIR criteria 2015; Supplementary Table S2. Categories and characteristics of the trophectoderm according to ASEBIR criteria 2015; Supplementary Table S3. Embryo classification on day 5 of development, according to ASEBIR criteria 2015, based on expansion grade, inner cell mass (ICM), and trophectoderm quality; Supplementary Table S4. Embryo classification on day 6 of development, according to ASEBIR criteria 2015, based on expansion grade, inner cell mass (ICM), and trophectoderm. Supplementary Table S5. Embryo development in ICSI and PICSI groups. Supplementary Table S6. Table data containing the number of transfers, pregnancies, miscarriages, and live births in fresh and deferred PICSI and ICSI transfers.

## Abbreviations

The following abbreviations are used in this manuscript:

AI	Artificial Intelligence
ASEBIR	Association for the Study of Reproductive Biology
ART	Assisted Reproductive Techniques
CB	Cavitated Blastocyst
CI	Confidence Interval
D5	Day 5
D6	Day 6
EB	Expanded Blastocyst
HA	Hyaluronic Acid
HB	Hatching Blastocyst
ICSI	Intracytoplasmic Sperm Injection
ICM	Inner Cell Mass
iHB	Initiating Hatching Blastocyst
IVF	In Vitro Fertilization
LBR	Live Birth Rate
m/rw	mean/receiving woman
MII	Metaphase II
OR	Odds ratio
PN	Pronuclei
*p*	p-value
PICSI	Physiological Intracytoplasmic Sperm Selection
RCT	Randomized Control Trial
TE	Trophectoderm
