# Supplementary material for: A Comprehensive Comparison of PICSI and ICSI Techniques Through a Triple-Blinded Trial: Effects on Embryo Quality, Cumulative Pregnancy Rate, and Live Birth Rate
Source: Biomedicines. 2025 May 1;13(5):1104. doi: 10.3390/biomedicines13051104 (PMC12108910; doi:10.3390/biomedicines13051104)
Supplement: Supplementary file 1 [file biomedicines-13-01104-s001.zip › Supplementary Table S1.pdf]

Supplementary Table S1. Categories and characteristics of embryonic inner cell mass according to ASEBIR criteria 2015.

**Category**	**Inner cell mass size (μm^2)**	**Cohesion**
A	3800-1900	Compact
B	3800-1900	Not compact
C	<1900	
D	Degeneration signs	Indifferent
Excluded	Degenerated	
