# Supplementary material for: A Comprehensive Comparison of PICSI and ICSI Techniques Through a Triple-Blinded Trial: Effects on Embryo Quality, Cumulative Pregnancy Rate, and Live Birth Rate
Source: Biomedicines. 2025 May 1;13(5):1104. doi: 10.3390/biomedicines13051104 (PMC12108910; doi:10.3390/biomedicines13051104)
Supplement: Supplementary file 1 [file biomedicines-13-01104-s001.zip › Supplementary Table S2.pdf]

Supplementary Table S2. Categories and characteristics of the trophectoderm according to ASEBIR criteria 2015.

**Category**	**Trophoectoderm description**
A	Homogeneous, united and full of cells
B	Homogeneous, less cells
C	Few cells
D	Degeneration signs
Excluded	Degenerated
