# Supplementary material for: A Comprehensive Comparison of PICSI and ICSI Techniques Through a Triple-Blinded Trial: Effects on Embryo Quality, Cumulative Pregnancy Rate, and Live Birth Rate
Source: Biomedicines. 2025 May 1;13(5):1104. doi: 10.3390/biomedicines13051104 (PMC12108910; doi:10.3390/biomedicines13051104)
Supplement: Supplementary file 1 [file biomedicines-13-01104-s001.zip › Supplementary Table S4.pdf]

Supplementary Table S4. Embryo classification on Day 6 of development, according to ASEBIR criteria 2015, based on expansion grade, ICM and TE quality.

**D6**			
**Expansion grade**	**ICM**	**TE**	**ASEBIR**
**Since 'starting expansion' Up to 'hatched'**	A	A	B
		B	
		C	C
		D	D
	B	A	B
		B	
		C	C
		D	D
	C	A	B
		B	
		C	C
		D	D
	D	A,B,C o D	D
Early blastocyst (Thick pellucid zone)			C
Morula		Excluded	
