# Supplementary material for: A Comprehensive Comparison of PICSI and ICSI Techniques Through a Triple-Blinded Trial: Effects on Embryo Quality, Cumulative Pregnancy Rate, and Live Birth Rate
Source: Biomedicines. 2025 May 1;13(5):1104. doi: 10.3390/biomedicines13051104 (PMC12108910; doi:10.3390/biomedicines13051104)
Supplement: Supplementary file 1 [file biomedicines-13-01104-s001.zip › Supplementary Table S5.pdf]

Supplementary Table S5. Embryo development in ICSI and PICSI groups.

		D5		D6		D5+D6	
		PICSI	ICSI	PICSI	ICSI	PICSI	ICSI
**Blastocyst rate (%)**		58.12	63.03	6.31	5.87	60.64	63.89
**Evolutionary embryo rate (%)**	**CB**	14.20	15.80	30.00	18.64	10.50	10.20
	**EB**	23.70	24.60	41.43	30.51	32.00	35.40
	**iHB**	8.60	7.60	5.71	5.08	15.40	13.10
	**HB**	0.00	0.10	1.43	0.00	0.20	0.30
**Good-quality embryo (%)**		68.27	63.47	35.50	30.77	54.20	50.70
**Non-viable embryo (%)**		2.84	3.79	27.94	23.81	13.60	13.50

* Percentages of Blastocyst rate on D6 refer only to blastocysts which arrive to blastocyst stage on that day.

** The percentages for D5+D6 are not simply the sum of the percentages for D5 and D6, as some D5 blastocysts degenerate by D6.
