# Supplementary material for: A Comprehensive Comparison of PICSI and ICSI Techniques Through a Triple-Blinded Trial: Effects on Embryo Quality, Cumulative Pregnancy Rate, and Live Birth Rate
Source: Biomedicines. 2025 May 1;13(5):1104. doi: 10.3390/biomedicines13051104 (PMC12108910; doi:10.3390/biomedicines13051104)
Supplement: Supplementary file 1 [file biomedicines-13-01104-s001.zip › Supplementary Table S6.pdf]

Supplementary Table S6. Table data containing number of transfers, pregnancies, miscarriages and live births in fresh and deferred PICSI and ICSI transfers.

<table>
<tr><td colspan="6">Fresh transfers PICSI</td></tr>
<tr><td colspan="6">104</td></tr>
<tr><td>Non pregnancy</td><td colspan="5">Pregnancy</td></tr>
<tr><td>27</td><td colspan="5">77</td></tr>
<tr><td rowspan="7"></td><td colspan="2">Miscarriage</td><td colspan="3">Pregnancies with live-birth</td></tr>
<tr><td colspan="2">22</td><td colspan="3">55</td></tr>
<tr><td>Clinical</td><td>Biochemical</td><td>1 live birth</td><td>2 live birth</td><td>3 live birth</td></tr>
<tr><td>13</td><td>9</td><td>45</td><td>9</td><td>1</td></tr>
<tr><td colspan="2" rowspan="2"></td><td colspan="3">Total live birth</td></tr>
<tr><td colspan="3">66</td></tr>
<tr><td colspan="5"></td></tr>
<tr><td colspan="6">Fresh ICSI transfers</td></tr>
<tr><td colspan="6">103</td></tr>
<tr><td>Non pregnancy</td><td colspan="5">Pregancy</td></tr>
<tr><td>30</td><td colspan="5">73</td></tr>
<tr><td></td><td colspan="2">Miscarriage</td><td colspan="3">Pregnancies with live-birth</td></tr>
<tr><td rowspan="5"></td><td colspan="2">14</td><td colspan="3">59</td></tr>
<tr><td>Clinical</td><td>Biochemical</td><td>1 livebirth</td><td>2 live birth</td><td>3 live birth</td></tr>
<tr><td>12</td><td>2</td><td>44</td><td>15</td><td>0</td></tr>
<tr><td colspan="2" rowspan="2"></td><td colspan="3">Total live birth</td></tr>
<tr><td colspan="3">74</td></tr>
<tr><td colspan="6">Deferred PICSI trasnfers</td></tr>
<tr><td colspan="6">99</td></tr>
<tr><td>Non pregnancy</td><td colspan="5">Pregnancy</td></tr>
<tr><td>48</td><td colspan="5">51</td></tr>
<tr><td rowspan="6"></td><td colspan="2">Miscarriage</td><td colspan="3">Pregnancies with live-birth</td></tr>
<tr><td colspan="2">19</td><td colspan="3">30</td></tr>
<tr><td>Clinical</td><td>Biochemical</td><td>1 livebirth</td><td>2 live birth</td><td>3 live birth</td></tr>
<tr><td>14</td><td>5</td><td>25</td><td>5</td><td>0</td></tr>
<tr><td colspan="2" rowspan="2"></td><td colspan="3">Total live birth</td></tr>
<tr><td colspan="3">35</td></tr>
<tr><td colspan="6">Deferred ICSI transfers</td></tr>
<tr><td colspan="6">99</td></tr>
<tr><td>Non pregnancy</td><td colspan="5">Pregnancy</td></tr>
<tr><td>50</td><td colspan="5">50</td></tr>
<tr><td rowspan="6"></td><td colspan="2">Miscarriage</td><td colspan="3">Pregnancies with live-birth</td></tr>
<tr><td colspan="2">21</td><td colspan="3">26</td></tr>
<tr><td>Clinical</td><td>Biochemical</td><td>1 live birth</td><td>2 live birth</td><td>3 live birth</td></tr>
<tr><td>14</td><td>7</td><td>24</td><td>2</td><td>0</td></tr>
<tr><td colspan="2" rowspan="2"></td><td colspan="3">Total live birth</td></tr>
<tr><td colspan="3">28</td></tr>
</table>
